# *Instrumento para Avaliação Formativa em Estágio Supervisionado de Enfermagem®:* elaboration and validity

**DOI:** 10.1590/0034-7167-2025-0004

**Published:** 2025-12-08

**Authors:** Tierle Kosloski Ramos, Silvana Bastos Cogo, Elisabeta Albertina Nietsche, Cléton Salbego, Vania Marli Schubert Backes, Edlamar Kátia Adamy, Etiane de Oliveira Freitas, Cristina Lavareda Baixinho

**Affiliations:** I Universidade Federal de Santa Maria. Santa Maria, Rio Grande do Sul, Brazil; II Centro Universitário Autônomo do Brazil. Curitiba, Paraná, Brazil; III Universidade Federal da Bahia. Salvador, Bahia, Brazil; IV Universidade do Estado de Santa Catarina. Florianópolis, Santa Catarina, Brazil; V Escola Superior de Enfermagem de Lisboa. Lisboa, Portugal

**Keywords:** Education, Nursing, Nursing Education Research, Performance, Academic, Clinical Clerkship, Students, Nursing., Educación en Enfermería, Investigación en Educación de Enfermería, Rendimiento Académico, Prácticas Clínicas, Estudiantes de Enfermería.

## Abstract

**Objectives:**

to develop and validate the *Instrumento para Avaliação Formativa de Estudantes de Enfermagem em Estágio Curricular Supervisionado* ^®^ content.

**Methods:**

a methodological study, carried out in two rounds using the modified e-Delphi technique. Its development was supported by an integrative literature review, official documents from the Ministry of Education, Federal Nursing Council, Brazilian Nursing Association and theoretical framework by Philippe Perrenoud.

**Results:**

in the first round, the instrument presented a Content Validity Index of 0.89, while in the second round, of 0.92. Reliability was 0.993. The instrument presents identification information, usage guidelines, four domains, 27 items, descriptive assessment by professors and supervisors, and student self-assessment.

**Conclusions:**

the instrument is a valid tool, allowing formative assessment. Its use, based on the guidelines established and based on the theoretical framework, encourages the procedural and regulatory assessment of student learning in supervised curricular internship.

## RESUMEN

**Objetivos:**

desarrollar y validar el contenido del *Instrumento para Avaliação Formativa de Estudantes de Enfermagem em Estágio Curricular Supervisionado* ^**®**^.

**Métodos:**

estudio metodológico, realizado en dos rondas mediante la técnica e-Delphi modificada. Su desarrollo se apoyó en una revisión bibliográfica integradora, documentos oficiales del Ministerio de Educación, el Consejo Federal de Enfermería, la Asociación Brasileña de Enfermería y el marco teórico de Philippe Perrenoud.

**Resultados:**

en la primera ronda, el instrumento presentó un Índice de Validez de Contenido de 0,89, mientras que en la segunda ronda fue de 0,92. La confiabilidad fue de 0,993. El instrumento presenta información de identificación, pautas de uso, cuatro dominios, 27 ítems, evaluación descriptiva del profesor y el supervisor, y autoevaluación del estudiante.

**Conclusiones:**

el instrumento es una herramienta válida que permite la evaluación formativa. Su uso, basado en las directrices establecidas y en el marco teórico, fomenta la evaluación procedimental y regulatoria del aprendizaje del estudiante en las prácticas curriculares supervisadas.

## INTRODUCTION

The Brazilian National Curricular Guidelines for the Undergraduate Course in Nursing (In Portuguese, *Diretrizes Curriculares Nacionais do Curso de Graduação em Enfermagem* - DCN-Enf), established in 2001, describe the general and specific competencies and skills required for nursing training. In addition to the theoretical and practical content developed during the course, it also recommends the mandatory completion of a supervised curricular internship (SCI) in general and specialized hospitals, outpatient clinics, the basic healthcare service network, and communities, which must be completed in the last two semesters, comprising at least 20% of the total course workload^(1)^. Nursing training in Brazil includes the development of internship activities in healthcare services mediated by nurses^(2)^.

SCI provides an opportunity to relive situations for nursing training, through the understanding and development of work processes, in addition to the acquisition and improvement of technical skills that underpin and permeate professional practice in health^(3)^. For the effective development of SCI, the commitment of those involved in these relationships is essential, which include students, supervising nurses, professors and managers^(4)^.

From this perspective, there is a need to adopt actions that ensure the quality standards of teaching, emanating from the DCN-Enf and compatible with the training of professionals capable of knowing, interpreting and intervening in the world in which they live^(5)^. Furthermore, there are many factors involved in academic performance, which is a complex construct of a multifactorial nature, especially associated with students’ personal history and experiences during graduation^(6)^.

Work and education intersect in the field of teaching SCI in nursing. Given this, there seems to be an indication to develop individualized clinical teaching strategies according to nursing students’ learning styles and to assess their holistic clinical competency^(7)^. With regard to learning assessment, the use of multiple assessments, with a combination of (in)direct measures, is recommended to ensure accuracy of students’ learning process and performance^(8)^.

In higher education in general, there are few studies that address assessment strategies and methods of constructing students’ knowledge. However, it is known that conventional practices, such as the use of tests, are still widely adopted by professors and educational institutions^(9)^. To this end, learning assessment should not only measure student performance, but also strengthen their autonomy and transcend improving the quality of teaching and learning^(10)^. Assessment does not represent the end of the learning process, but rather an ongoing means of assessing progress towards established objectives. It highlights areas of success and those that require additional attention, enabling adjustments to educational plans to effectively address identified challenges^(11)^.

Assessment does not represent the end of the learning process, but rather an ongoing means of assessing progress towards established objectives. It highlights areas of success and those that require additional attention, enabling adjustments to educational plans to effectively address identified challenges^(12)^.

Given this scenario, it is believed that the development and validity of an instrument to assess students’ teaching-learning process during SCI with a panel of experts can contribute to strengthening assessment. Moreover, this initiative will promote an approach aligned with the DCN-Enf requirements.

## OBJECTIVES

To develop and validate the *Instrumento de Avaliação Formativa de Discentes de Enfermagem em Estágio Curricular Supervisionado* (IAFEE-ECS^®^) content.

## METHODS

### Ethical aspects

This study complied with Resolutions 466/2012^(13)^ and 510/2016^(14)^. The research was forwarded for consideration by the *Universidade Federal de Santa Maria* Research Ethics Committee and was approved on July 12, 2022.

### Study design, period and location

This is a methodological study, developed between January and September 2023, guided by the Standards for QUality Improvement Reporting Excellence 2.0: revised publication guidelines from a detailed consensus process^(15)^. IAFEE-ECS^®^ was based on Philippe Perrenoud’s theoretical framework for formative assessment^(12)^, and for validity, the modified e-Delphi technique was used in two rounds, which is characterized by the identification of consensus and disagreement on the topic, interconnection and potential for integration of knowledge^(16)^.

### Population and inclusion and exclusion criteria

The study population consisted of nurse researchers with expertise in nursing education, who were located through a search on the *Lattes* Platform and the Directory of Research Groups in Brazil of the Brazilian National Council for Scientific and Technological Development. It is noteworthy that, in January 2023, there were 70 research groups in nursing that include nursing education in their line of research. Course coordinators and/or professors directly involved in SCI of nursing courses at public Higher Education Institutions (HEIs) were also included. Between January and February 2023, a mapping was carried out on the Ministry of Education and Culture’s (In Portuguese, *Ministério da Educação e Cultura* - MEC) website to list HEIs with active undergraduate nursing courses of federal and/or state public administrative category, totaling 55 federal and 36 state institutions. From this information, it was possible to access HEIs’ websites to obtain the email addresses of course coordinators and to invite them to participate in the research and/or indicate the professor responsible for SCI at the institution. To expand the study sample, the snowball technique was used, which is characterized by participant selection based on the indication of previous participants^(17)^.

The inclusion criteria adopted for nurse researchers were to be a nurse, have teaching experience and scientific publications in nursing training. For coordinators and/or professors, it was necessary to be a nurse, work as a professor linked to a HEI, be a course coordinator or have experience as a professor in SCI. For both groups, those who responded to the instrument after the established deadline of 20 days and/or who did not establish communication with the researchers after three attempts to contact them were excluded.

### Study protocol

The development of the instrument emerged from the results of previous research^(18-20)^, which aimed to analyze SCI in nursing from the perspective of students, graduates, internship supervisors, faculty advisors and managers in nursing training. Such research allowed us to identify the limitations related to students’ assessment process in SCI and to indicate relevant and necessary criteria for the structuring of an instrument capable of assessing the expected skills more comprehensively. In addition to this, an integrative literature review was carried out aiming to understand evidence of the evaluative phenomenon in nursing training, in which searches were carried out in the following databases: Latin American and Caribbean Literature in Health Sciences, Scopus, PubMed, Excerpta Medica dataBASE and Education Resources Information Center. The search took place between April and May 2023. Initially, 3,598 articles were found, which were subsequently analyzed according to inclusion and exclusion criteria, and 82 articles comprised the final sample of the review. Subsequently, official documents from the MEC, the Federal Nursing Council (In Portuguese, *Conselho Federal de Enfermagem* - COFEN) and the Brazilian Nursing Association were consulted^(21-23)^. It is worth noting that, in addition to the current DCN-Enf, the new DCN-Enf drafts were also used to prepare the instrument. With this data in hand, the first version of IAFEE-ECS^®^ domains and items was prepared.

After IAFEE-ECS^®^ was developed, the validity stage began with the panel of experts. The research was conducted online, seeking participants from different regions of the country, in order to allow a broad range of experts - nurse researchers and course coordinators and/or professors directly involved in SCI -, as it is characterized as the first stage towards the dissemination of knowledge in development. Expanding the data collection scenario in validity studies is relevant as different people, with their perceptions, life and work contexts, are included in a sample.

This stage was carried out from June to September 2023 in two rounds. Invitations were sent to 260 experts via email containing a brief summary of the research, its objectives, its method and a link to access the Informed Consent Form. Sixty-seven experts agreed to participate in the study, who were informed about the deadline established for returning the validity instrument, and who indicated new experts who met the study selection criteria. After acceptance, participants received an email with the link to access Google Forms^®^ containing IAFEE-ECS^®^ as well as guidelines for carrying out its validity.

In the first round, 24 experts returned to validate the instrument, presenting suggestions for improvements. For each chunk of IAFEE-ECS^®^, the clarity, relevance, objectivity, precision, variety, modality, behavior, breadth, balance, typicality, credibility and simplicity criteria were assessed^(24)^. The instrument was presented in the form of a Likert-type scale, composed of four points: 1 - Inadequate; 2 - Slightly Adequate; 3 - Adequate; 4 - Totally Adequate. For items assessed with a score of 1 or 2, experts were asked to provide suggestions for changes and/or addition or exclusion of items.

After consensus meetings among the authors, IAFEE-ECS^®^ assessment chunks that presented a Content Validity Index (CVI) lower than 0.80 were excluded, and other items were changed according to experts’ opinion. Subsequently, the second round was initiated, through controlled feedback to the experts who participated in the first round.

In the second round, the revised IAFEE-ECS^®^ was made available via a Google Forms^®^ link, indicating the excluded or reformulated items. In relation to the excluded content, experts were asked to indicate whether or not they agreed with this exclusion. The reformulated chunks were assessed according to the same psychometric criteria as the previous round. It is worth noting that no new participants were included in this round. The experts were given a 20-day deadline to respond to the second round. Those who did not respond were sent weekly reminders for 15 days, and the data collection for the second round was completed with the participation of 12 experts.

### Analysis of results, and statistics

The data were stored in spreadsheets and analyzed with the support of the Statistical Package for the Social Sciences statistical program. The instrument reliability and internal consistency assessment was analyzed using Cronbach’s alpha coefficient^(25)^. Agreement of over 80% was established for each chunk and item submitted for validity^(26)^. It is important to note that there is no universal agreement on what the level of consensus should be for a Delphi study, and this should be a decision made by the researcher. Thus, the overall validity of psychometric criteria, as well as the instrument chunks and items, was assessed by CVI. In the present study, a CVI ≥ 0.80 was considered adequate^(16)^. In the analysis process, experts’ comments and suggestions were also considered, with a view to including qualitative elements, especially with the aim of providing understandable information about the instrument.

## RESULTS

The instrument was developed based on the theoretical elements of formative assessment proposed by Perrenoud’s framework^(12)^, which aims to enable assessment to be conducted during SCI, and can be used by professors, supervising nurse and student, enabling their self-assessment throughout the period. To this end, the instrument should serve as a means to individualize the course of the teaching-learning process throughout internship.

The first version of IAFEE-ECS^®^ presented a structure composed of five assessment chunks: 1 - header with student identification data, HEI and internship location; 2 - presentation and guidelines for using the instrument; 3 - the four instrument domains with their respective items: I - domain: transversal skills and abilities identified/evidenced in the development of supervised curricular internship, containing nine items; II - domain: nursing care and assistance in human healthcare, containing ten items; III - domain: nursing management in services, containing six items; IV - domain: education, investigation/research in nursing and health, containing five items; 4 - below each domain, space was made available for the evaluator and the student to write a descriptive report, aiming at their self-assessment; 5 - at the end of IAFEE-ECS^®^, a formula was made available to carry out the final formative assessment.

In the instrument columns, located on the right, the evaluator has the opportunity to assign a score (point) from 0 to 10 for each item in each formative assessment, considering students’ development in the internship field, the same process being carried out by student in their self-assessment. At the end of each domain, the sum of the scores for each item must be recorded. The sum of the scores obtained in each domain, divided by the number of items in the instrument, will be the result of each formative assessment (FA1 and FA2). To obtain students’ summative assessment, the simple arithmetic mean of formative assessments must be calculated. To do so, the sum of scores obtained in formative assessments 1 and 2 (FA1 + FA2) must be calculated and the result divided by 2, resulting in the final average.

Twenty-four experts aged between 33 and 68 participated in IAFEE-ECS^®^ content validity, of which 22 (91.7%) were female and two (8.3%) were male. All participants had a degree in nursing; of these, 15 (62.5%) had a doctoral degree, seven (29.2%) had a postdoctoral degree, and two (8.3%) had a master’s degree. As for professional experience, all of them are working in higher education nursing teaching, but 23 (95.8%) reported previous experience in healthcare, and 14 (58.3%) in management positions. Regarding the areas of activity in the research, 13 (54.2%) had a specialization in medical-surgical nursing, six (25%) in public health nursing, four (16.7%) in obstetric nursing, and one (4.2%) in contagious disease nursing.

Concerning expert panel characterization, there was a predominance of experts with a doctoral degree, with previous professional experience in assistance and who have worked and/or work in the SCI discipline.

In the first round, 24 experts participated. The psychometric criteria Scale-level Content Validity Index (S-CVI) was 0.89, and Cronbach’s alpha was 0.993 (Table 1). There was no consensus among experts regarding the title of domain I (CVI 0.79) and also regarding items 13 (CVI 0.73), 18 (CVI 0.79) and 30 (CVI 0.79) (Table 1). To proceed with the second round of validity, the wording of the title of domain I was changed; item 13 was excluded because it was included in the wording of the previous item; and items 18 and 30 were excluded as suggested by experts, due to the impossibility of using them in assessment of students’ practice.

**Table 1:** Items that make up the *Instrumento de Avaliação Formativa de Discentes de Enfermagem em Estágio Curricular Supervisionado*, Santa Maria, Rio Grande do Sul, Brazil, 2023

ASSESSED ITEMS			
	CVI		
Identification elements	Round 1	Round 2	ALPHA
Instrument title.	0.91	-	0.932
Identification data.	0.89	1.00	0.912
**Instrument items**			
***Domínio I: Competências e habilidades transversais identificadas/evidenciadas no desenvolvimento do estágio curricular supervisionado***	0.79	1.00	0.977
*Demonstra domínio do conhecimento teórico e científico sobre os temas que emergem das situações práticas vivenciadas no campo de estágio.*	0.85	-	0.964
*Demonstra conhecimento das principais políticas e legislações de saúde.*	0.83	-	0.966
*Compreende a ética e os princípios bioéticos, respeitando a dignidade, a autonomia e a privacidade dos indivíduos.*	0.97	-	0.918
*Compreende as diversidades de gênero, raciais, culturais, sociais e econômicas dos indivíduos e comunidades.*	0.96	-	0.934
*Capacidade para trabalhar em ambientes complexos e dinâmicos, adaptando-se a mudanças e novas situações, sendo capaz de resolver problemas que surgem no cotidiano do trabalho.*	0.91	-	0.955
*Demonstra autonomia na prática de enfermagem dentro dos limites de sua formação e competência.*	0.97	-	0.932
*Trabalha de forma colaborativa com a equipe de enfermagem e interprofissional.*	0.97	-	0.952
*Comunica-se, de forma clara, efetiva e respeitosa, com pacientes, familiares e profissionais de saúde, sendo capaz de utilizar formas alternativas de comunicação.*	0.96	-	0.938
*Demonstra habilidades na execução e registro do Processo de Enfermagem, apoiado nos Sistemas de Linguagem Padronizada de enfermagem.*	0.93	-	0.963
***Domínio II: Assistência e cuidado de enfermagem na atenção à saúde humana***	0.88	-	0.978
*Demonstra conhecimento nos principais procedimentos, técnicas e tecnologias utilizados na prática de enfermagem.*	0.97	-	0.934
*Demonstra habilidades na execução de procedimentos e técnicas seguras, além do uso de tecnologias no cuidado.*	0.88	-	0.974
*Identifica as patologias e condições de saúde prevalentes na população, (co)relacionando aos determinantes sociais de saúde.*	0.84	-	0.954
*Demonstra reconhecer o processo de saúde-doença prevalentes*	0.73	-	0.977
*Desenvolve a prática de enfermagem pautada no pensamento crítico, raciocínio clínico e baseado em evidências.*	0.94	-	0.959
*Presta assistência de enfermagem de forma segura e eficaz.*	0.89	-	0.976
*Demonstra responsabilidade e compromisso com a melhoria contínua da qualidade do cuidado.*	0.95	-	0.919
*Presta cuidados de enfermagem compatíveis com as diferentes necessidades apresentadas pelo indivíduo, família e comunidade.*	0.96	-	0.984
*Compreende a natureza humana em suas dimensões e fases evolutivas.*	0.77	-	0.985
*Demonstra reconhecer o funcionamento da Rede de Atenção à Saúde, em seus diferentes níveis, na perspectiva da integralidade da assistência.*	0.90	-	0.986
***Domínio III: Gestão de enfermagem nos serviços***	0.99	-	0.948
*Realiza análise situacional do processo de trabalho para planejar e propor intervenções.*	0.93	-	0.990
*Organiza e direciona recursos materiais, dimensionamento de pessoal e de infraestrutura para a realização do trabalho de enfermagem.*	0.97	-	0.928
*Atua junto à equipe de enfermagem e multiprofissional com relacionamento interpessoal adequado, comunicando-se de modo efetivo, interagindo e propondo resolução diante dos problemas encontrados no campo profissional.*	0.92	-	0.970
*Utiliza instrumentos que qualificam o cuidado (escala de dimensionamento de pessoal, escalas para avaliação da complexidade assistencial, escalas de avaliação da condição atual e/ou de risco do paciente).*	0.89	-	0.984
*Utiliza o Processo de Enfermagem, incluindo os Sistemas de Linguagem Padronizadas de enfermagem.*	0.91	-	0.996
*Desenvolve ações de liderança da equipe de enfermagem, promovendo integração, atualização e qualificação.*	0.88	-	0.968
***Domínio IV: Educação, investigação/pesquisa em enfermagem e saúde***	0.98	-	0.928
*Disposição para o aprendizado contínuo e busca por atualização. Durante o estágio o aluno realizou cursos e/ou participou de eventos.*	0.85	-	0.982
*Utiliza novas tecnologias de educação, informação e comunicação com a equipe de saúde e/ou pacientes e família.*	0.85	-	0.983
*Capacidade de planejar, implementar e avaliar estratégias educativas em saúde pautadas no diálogo e valorização dos saberes dos envolvidos.*	0.87	-	0.979
*Capacidade de elaborar, executar e avaliar projetos de intervenção profissional.*	0.80	-	0.987
*Planeja, implementa e avalia os programas de formação e qualificação dos trabalhadores.*	0.75	-	0.991
**Guiding elements for using the instrument**			
Considering: the presentation and guidelines on the use of the instrument	0.86	1.00	0.955
Guidelines and organization of assessment to obtain the final average	0.83	1.00	0.967
Considering the “self-assessment” field (intended for the student), located at the end of each domain.	0.84	1.00	0.961
Considering the “observations/comments” field (intended for the professor and preceptor), located at the end of each domain.	0.96	1.00	0.917
As for the instrument closure.	0.84	1.00	0.985
**S-CVI/Ave**	**0.89**	**0.92**	
**Instrument Cronbach’s alpha**			**0.993**

*CVI - Content Validity Index; S-CVI/AVE - Scale-level Content Validity Index/Average.*

In the second assessment round, the instrument’s S-CVI obtained 0.92. It is observed that CVI and Cronbach’s alpha were not presented by psychometric criteria in the second Delphi round, since only items with CVI below 0.80 were submitted for assessment by experts (Table 2).

**Table 2:** Instrumento de Avaliação Formativa de Discentes de Enfermagem em Estágio Curricular Supervisionado Content Validity Index and Reliability based on psychometric criteria, Santa Maria, Rio Grande do Sul, Brazil, 2023

Psychometric criteria	CVI	Cronbach’s alpha
	Delphi 1	Delphi 1
Clarity	0.83	0.823
Relevance	0.93	0.895
Objectivity	0.83	0.963
Precision	0.84	0.962
Variety	0.91	0.921
Modality	0.92	0.935
Behavior	0.89	0.967
Breadth	0.91	0.921
Balance	0.91	0.926
Typicality	0.90	0.955
Credibility	0.93	0.938
Simplicity	0.88	0.955
S-CVI	**0.89**	**0.993**

*CVI - Content Validity Index; S-CVI - Scale-level Content Validity Index.*

Therefore, regarding IAFEE-ECS^®^ reliability, its internal consistency, through Cronbach’s alpha coefficient, obtained values higher than 0.823 in the 12 psychometric criteria of the instrument, demonstrating high internal consistency (Table 2).

According to experts’ comments and suggestions, there were specific changes in the following chunks: identification data (the “registration” field was added, a field to mark one of the following three options - “primary healthcare”, “secondary healthcare” and “tertiary healthcare” - and a field for filling in the “workload carried out”); adjustments to the wording of the text “presentation and guidelines on the instrument use” and in the text “instrument application”, in which a brief presentation of each domain was added; the field intended for “self-assessment”, as well as the “field to describe observations/comments regarding the 1^st^ and 2^nd^ formative assessment, if necessary”, which were at the end of each domain with only one space to be filled in, which were relocated to the end of the instrument and subdivided into two assessment moments, as suggested by experts, aiming to monitor students’ progress; at the end of the instrument, a field was included to indicate who participated in formative assessment, such as “advisory professor”, “supervising nurse” and “student”, since it can be used both by students, for self-assessment, and by advisory professors and also by preceptor nurses.

After the validity process with adaptation of the instrument according to experts’ opinion, its final version was established. Subsequently, IAFEE-ECS^®^ was submitted to the Copyright Registration by the Brazilian Book Chamber, receiving ISBN nº 978-65-00-93619-3. The referred version is available at the link: https://www.ufsm.br/cursos/pos-graduacao/santa-maria/ppgenf/produtos-tecnico-tecnologicos.

## DISCUSSION

In order to achieve more effective pedagogy, it is essential to know which skills and abilities students are to be guided towards as well as the necessary paths. In addition, there is a need to identify the means available to observe the domains achieved or in the process of being acquired, the work methods, attitudes, mental functioning and how students are to be intervened with through learning regulations. Most teaching methods are not designed by a differentiated pedagogy resulting from formative assessment. To move in this direction, a large investment in the creation or adaptation of teaching tools must be accepted^(12)^.

The DCN-Enf emphasizes that students’ formative assessment must be based on curricular content, skills and competencies, seeking the development of criticism, reflection, ethics, among other fundamental elements^(1)^. From Perrenoud’s perspective, assessment is not an objective in itself, but a means of verifying whether students have acquired the necessary knowledge and, based on this, managing teaching situations. However, it is important to emphasize that learning is never linear, as it is carried out through tests, trials and errors, suggestions and counter-suggestions, complementary explanations, review of basic notions, and work on the meaning of the task or self-confidence^(12)^. Based on this, nursing training must enable the development of transversal skills, in order to prepare professionals not only for efficient professional practice, but also for the dynamic and multifaceted challenges of healthcare services.

Knowledge about health policies and legislation, ethics and bioethics, and the understanding of gender, racial, cultural, social and economic diversity of individuals and communities are fundamental and interconnected elements that are present in nurses’ daily work, and are provided for in the DCN-Enf^(1)^. When faced with real events, interns are encouraged to reflect on the different situations that arise in the context of health work, leading them to plan their actions and only later implement them^(27)^. Given this, training requires not only a theoretical understanding of these elements, but also the application of this understanding in their interaction in clinical practice with patients and interdisciplinary teams.

The DCN-Enf’s specific skills and competencies include the ability to diagnose and solve health problems, communicate, make decisions, intervene in the work process, work as a team and deal with constantly changing situations^(1)^. Establishing dialogue with patients and healthcare teams or expressing opinions coherently are difficulties faced by students during their training, which highlights the need to strengthen communication skills^(28)^. To this end, professionals must have solid interpersonal skills to work effectively, fostering healthy relationships with the multidisciplinary team and proposing solutions to professional challenges.

Transversal competencies seek to assess whether students can interrelate theory and practice to execute the Nursing Process (NP)^(29)^. In training, the development of students’ critical and reflexive skills is essential for them to identify pathologies, health conditions, prevalence, incidence, outline indicators and relate all these findings from their practice to the social determinants of health at different levels of care, including primary, secondary and tertiary.

Formative assessment provides professors with more precise and qualitative information about students’ learning processes, attitudes and acquisitions^(12)^. To implement the NP, professors and supervisors must help students broaden their view of the population’s health and illness conditions. During this learning process, students’ critical thinking, clinical reasoning, and potential to combine all findings from clinical practice with the best scientific evidence are assessed.

Formative assessment is characterized as a device for individualizing the training process and differentiating interventions with each student, seeking to value their specific needs, constituting a differentiated intervention. Therefore, the assessment can never be analyzed in itself, but rather as a component of a system of action^(12)^. IAFEE-ECS^®^ proposes that nursing professionals not only need to assess technical knowledge, but also students’ scientific potential. Experiencing practice should go beyond identifying needs, as it is the time to use evidence-based practice to develop nursing planning aimed at implementing safe and effective care practices for individuals, families and communities. Furthermore, there is the need to (re)know the Healthcare Network as well as a vision based on comprehensiveness of care.

IAFEE-ECS^®^ was developed with the aim of providing elements to assess skills related to assessment, planning, implementation of interventions, organization and directing of material resources, staffing and infrastructure aimed at qualifying care. However, training has weaknesses in the development of management skills, with little depth in disciplines throughout the course or addressed in specific situations and in isolation in the curricula. Managing care, the nursing team and healthcare services is not an easy task, as it challenges nurses on a daily basis. To this end, it was identified that undergraduates recognize the importance of management in training and emphasize that the topic should be addressed from the first year of graduation^(30)^.

Formative assessment allows professors and supervisors to have a more precise idea of students’ intellectual functioning as well as their ways of learning and their level of competency^(12)^. In this sense, it is essential that professionals are constantly updated to scientifically support their work process. Therefore, the use of care qualification instruments, such as staffing scales and classification systems, is essential, but their effectiveness requires an in-depth understanding of the context in which they are applied. The application of NP reflects professional maturity, but the effectiveness of these practices lies in their cohesive integration with clinical reality.

Interns recognize the importance of personal commitment during SCI, since the development of their decision-making process requires the mobilization of attributes necessary for critical reasoning and facing dilemmas to decide what is considered best for the subject and for the society to which they belong^(27)^. Nursing training, based on continuous learning, is a crucial pillar in the face of the evolving health dynamics. Thus, the search for qualification (courses, participation in events, research and extension activities) indicates a personal interest in developing oneself and a professional interest in staying up to date.

The incorporation of new technologies into communication with staff and patients, although essential, requires a strategic approach that goes beyond mere digital inclusion. The ability to plan, implement and assess health education strategies, based on dialogue and appreciation of knowledge, is a crucial skill. However, its effectiveness is intrinsically linked to a deep understanding of the work environment’s specific needs. The ability to design, execute and assess professional intervention projects is undoubtedly relevant, but it requires that these initiatives go beyond the conceptual level and achieve an effective impact on clinical practice.

Often, only at the end of a study cycle does the assessment take place, resulting in short periods of time to effectively assist students in their learning, leading to short-term decisions that are sometimes difficult to reverse^(12)^. Regarding the use of IAFEE-ECS^®^ by professors, supervising nurses and students, it is recommended that it not be used only at the end of the assessment process, with the sole purpose of assigning a grade to students. Therefore, it is recommended that its use accompany the assessment process from the beginning of SCI, in order to serve as a tool for the continuous diagnostic assessment of learning. The adoption of this system can contribute to the identification of problems and difficulties, and monitoring of the training process through feedback on student performance, allowing interventions to improve learning.

It is important to train students to regulate their own thought and learning processes^(12)^. The instrument can also be used for self-regulation, providing students with the opportunity to look at their skills and abilities and plan, together with their supervising nurses and professors, their continuous development.

Adopting learning assessment from a training perspective encourages the development of a reflection on alternatives to traditional assessment, especially in the sense of an observation based on objectives or criteria of mastery of what is intended to be assessed. Although nothing changes overnight in the training process, (re)thinking assessment in nursing training also encourages HEIs to reconsider the entire teaching process, given that both are directly correlated.

In this study, students were considered as the center of the clinical practice teaching process, giving full consideration to individual learning style. It could ensure nursing students’ learning interest and improve the knowledge receiving effect^(7)^. This feedback between students, supervisors, professors and educational institutions is important so that the assessment process achieves its objective as a strategy for improving student performance^(31)^.

### Study limitations

The research is limited by including participants from public HEIs in nursing, not including private institutions.

### Contributions to nursing

As a contribution to nursing, it is believed that an instrument based on the DCN-Enf, official COFEN documents, as well as national and international literature, supported by a theoretical framework in the area of education that defends a differentiated pedagogy based on a formative assessment, can serve as a means to support learning assessment during SCI, and can be used by both professors and supervising nurses and by the students themselves for their self-regulation.

## CONCLUSIONS

IAFEE-ECS^®^, after its validity with experts, presents domains, items and guiding elements for its use, demonstrating its possibility for a training, procedural and self-regulatory assessment of students who are carrying out SCI. The study has the potential to stimulate new discussions about assessment processes not only during SCI period, but throughout nursing training, from the perspective of an innovative pedagogy that includes assessment at the center of pedagogical processes with the aim of serving as a means of regulating learning and not only as a specific assessment with the objective of complying with the obligation to grade students. It is recommended that the instrument be applied in different scenarios, including primary, secondary and tertiary care, by all actors involved in SCI, in order to verify its applicability in internship practice.

## Data Availability

The research data are available only upon request.
