# Supplementary material for: Instrumento para Avaliação Formativa em Estágio Supervisionado de Enfermagem®: elaboration and validity
Source: Rev Bras Enferm. 2025 Dec 8;78(5):e20250004. doi: 10.1590/0034-7167-2025-0004 (PMC12707535; doi:10.1590/0034-7167-2025-0004)
Supplement: Translation

# Translation: Instrumento para Avaliação Formativa em Estágio Supervisionado de Enfermagem^®^: elaboração e validação

**DOI:** 10.1590/0034-7167-2025-0004pt

**Authors:** Tierle Kosloski Ramos, Silvana Bastos Cogo, Elisabeta Albertina Nietsche, Cléton Salbego, Vania Marli Schubert Backes, Edlamar Kátia Adamy, Etiane de Oliveira Freitas, Cristina Lavareda Baixinho

**Affiliations:** I Universidade Federal de Santa Maria. Santa Maria, Rio Grande do Sul, Brasil; II Centro Universitário Autônomo do Brasil. Curitiba, Paraná, Brasil; III Universidade Federal da Bahia. Salvador, Bahia, Brasil; IV Universidade do Estado de Santa Catarina. Florianópolis, Santa Catarina, Brasil; V Escola Superior de Enfermagem de Lisboa. Lisboa, Portugal

**Keywords:** Educação em Enfermagem, Pesquisa em Educação de Enfermagem, Desempenho Acadêmico, Estágio Clínico, Estudantes de Enfermagem.

## Abstract

**Objetivos:**

elaborar e validar o conteúdo do Instrumento para Avaliação Formativa de Estudantes de Enfermagem em Estágio Curricular Supervisionado^**®**^.

**Métodos:**

estudo metodológico, realizado em duas rodadas por meio da técnica e-Delphi modificada. Sua elaboração se sustentou a partir de revisão integrativa de literatura, documentos oficiais do Ministério da Educação, Conselho Federal de Enfermagem, Associação Brasileira de Enfermagem e referencial teórico de Philippe Perrenoud.

**Resultados:**

na primeira rodada, o instrumento apresentou Índice de Validade de Conteúdo de 0,89, enquanto que, na segunda rodada, de 0,92. A confiabilidade foi de 0,993. O instrumento apresenta informações de identificação, orientações de uso, quatro domínios, 27 itens, avaliação descritiva pelo docente e supervisor, e autoavaliação do estudante.

**Conclusões:**

o instrumento é uma ferramenta válida, permitindo avaliação formativa. Sua utilização, a partir das orientações estabelecidas e fundamentadas no referencial teórico, incentiva a avaliação processual e de regulação da aprendizagem do estudante no estágio curricular supervisionado.

## INTRODUÇÃO

As Diretrizes Curriculares Nacionais do Curso de Graduação em Enfermagem (DCN-Enf), instituídas em 2001, descrevem as competências e habilidades gerais e específicas requeridas para a formação do enfermeiro. Além dos conteúdos teóricos e práticos desenvolvidos no transcorrer do curso, também preconiza a obrigatoriedade da realização do estágio curricular supervisionado (ECS) em hospitais gerais e especializados, ambulatórios, rede básica de serviços de saúde e comunidades, que deve ser realizado nos dois últimos semestres, compreendendo, no mínimo, 20% da carga horária total do curso^(1)^. A formação do enfermeiro no Brasil prevê o desenvolvimento de atividades de estágio nos serviços de saúde mediadas pelo enfermeiro^(2)^.

O ECS oportuniza reviver situações para a formação do enfermeiro por meio da compreensão e desenvolvimento de processos de trabalho, além da aquisição e aperfeiçoamento de habilidades técnicas que fundamentam e permeiam o exercício profissional em saúde^(3)^. Para o desenvolvimento efetivo do ECS, é indispensável o compromisso dos envolvidos nessas relações, que incluem o estudante, o enfermeiro supervisor, o docente e os gestores^(4)^.

Nessa perspectiva, há necessidade de adoção de ações que assegurem os padrões de qualidade de ensino, emanados das DCN-Enf e compatíveis com a formação de profissionais capazes de conhecer, interpretar e intervir no mundo em que vivem^(5)^. Ademais, existem muitos fatores envolvidos no desempenho acadêmico, que é um construto complexo de caráter multifatorial, especialmente associado à história pessoal e às experiências do estudante durante a graduação^(6)^.

O trabalho e a educação se interpenetram no campo do ensino do ECS na enfermagem. Por isso, parece haver uma indicação sobre desenvolver estratégias de ensino clínico individualizadas de acordo com os estilos de aprendizagem dos estudantes de enfermagem e avaliar a sua competência clínica holística^(7)^. No que diz respeito à avaliação da aprendizagem, recomenda-se o uso de avaliações múltiplas, com uma combinação de medidas (in)diretas para garantir a precisão do processo de aprendizagem e do desempenho do estudante^(8)^.

Na educação superior em geral, existem poucos estudos que abordam as estratégias de avaliação e os métodos de construção do conhecimento dos estudantes. Entretanto, sabe-se que práticas convencionais, como o uso de provas, ainda são amplamente adotadas por professores e instituições de ensino^(9)^. Para tanto, a avaliação da aprendizagem não deve medir apenas o desempenho dos estudantes, mas também fortalecer a sua autonomia e transcender a melhoria da qualidade do ensino-aprendizagem^(10)^. A avaliação não representa o fim do processo de aprendizado, mas sim um meio contínuo para avaliar o progresso em relação aos objetivos estabelecidos. Ela destaca áreas de sucesso e aquelas que exigem atenção adicional, possibilitando ajustes nos planejamentos educacionais para abordar eficazmente os desafios identificados^(11)^.

A partir do exposto, é relevante destacar que a questão mencionada está alinhada com o conceito de avaliação formativa proposto por Philippe Perrenoud. Esta abordagem é caracterizada como uma prática de avaliação contínua, cujo propósito é aprimorar as aprendizagens, e envolve uma análise dos avanços e do desempenho dos estudantes através da administração das aprendizagens. Seu intuito é avaliar o progresso já alcançado por cada estudante e o que ainda resta a percorrer, com a finalidade de intervir para aprimorar os processos em andamento. Perrenoud defende a autorregulação, visando estimular o autodesenvolvimento e a autoaprendizagem do estudante. Essa ação pode fortalecer as habilidades do sujeito em gerenciar seus projetos, avanços e estratégias diante das atividades e desafios^(12)^.

Diante desse cenário, acredita-se que a elaboração e validação de um instrumento para avaliar o processo de ensino-aprendizagem dos estudantes durante o ECS com um painel de especialistas pode contribuir para fortalecer a avaliação. Além disso, essa iniciativa promoverá uma abordagem alinhada às exigências das DCN-Enf.

## OBJETIVOS

Elaborar e validar o conteúdo do Instrumento para Avaliação Formativa de Estudantes de Enfermagem em Estágio Curricular Supervisionado (IAFEE-ECS^®^).

## MÉTODOS

### Aspectos éticos

O presente estudo respeitou as Resoluções nº 466/ 2012^(13)^ e nº 510/ 2016^(14)^. A pesquisa foi encaminhada para apreciação pelo Comitê de Ética em Pesquisa com seres humanos da Universidade Federal de Santa Maria, sendo aprovado no dia 12 de julho de 2022.

### Desenho, período e local do estudo

Trata-se de estudo metodológico, desenvolvido no período entre janeiro e setembro de 2023, norteado pelas diretrizes *Standards for QUality Improvement Reporting Excellence 2.0: revised publication guidelines from a detailed consensus process* ^(15)^. O IAFEE-ECS^®^ foi fundamentado pelo referencial teórico de avaliação formativa de Philippe Perrenoud^(12)^, e para validação, foi utilizada a técnica e-Delphi modificada em duas rodadas, a qual se caracteriza pela identificação de consenso e discordância sobre a temática, interligação e potencial para integração dos conhecimentos^(16)^.

### População e critérios de inclusão e exclusão

A população do estudo foi composta por pesquisadores enfermeiros com *expertise* na área da formação em enfermagem, que foram localizados por meio de busca na Plataforma *Lattes* e no Diretório dos Grupos de Pesquisa no Brasil do Conselho Nacional de Desenvolvimento Científico e Tecnológico. Destaca-se que, em janeiro de 2023, havia 70 grupos de pesquisa na área da enfermagem que contemplam em sua linha de pesquisa a formação de enfermeiros. Também foram incluídos os coordenadores de curso e/ou docentes diretamente envolvidos no ECS de cursos de enfermagem de Instituições de Ensino Superior (IES) públicas. Realizou-se entre os meses de janeiro e fevereiro de 2023 um mapeamento no site do Ministério da Educação e Cultura (MEC) para listar as IES com cursos de graduação em enfermagem ativos de categoria administrativa pública federal e/ou estadual, totalizando 55 instituições federais e 36 estaduais. A partir dessas informações, foi possível acessar os sites das IES para obter os endereços de e-mail das coordenações dos cursos e realizar o convite para participação na pesquisa e/ou indicação do docente responsável pelo ECS na instituição. Para ampliar a amostra do estudo, utilizou-se a técnica de bola de neve, que caracteriza-se pela seleção de participantes por indicação de participantes prévios^(17)^.

Foram adotados como critérios de inclusão para os enfermeiros pesquisadores ser enfermeiro, possuir experiência docente e publicações científicas na área de formação em enfermagem. Para os coordenadores e/ou docentes, era necessário ser enfermeiro, atuar como docente vinculado a uma IES, ser coordenador de curso ou ter vivenciado como docente o ECS. Para ambos os grupos, foram excluídos aqueles que responderam ao instrumento fora do prazo estabelecido de 20 dias e/ou que não estabeleceram comunicação com as pesquisadoras após três tentativas de contato.

### Protocolo do estudo

A elaboração do instrumento emerge de resultados de pesquisas anteriores^(18-20)^, que tiveram o objetivo de analisar o ECS em enfermagem na perspectiva dos discentes, egressos, supervisores de estágio, docentes orientadores e gestores na formação do enfermeiro. Tais pesquisas permitiram identificar as limitações relacionadas ao processo avaliativo dos estudantes em ECS e indicar critérios relevantes e necessários para a estruturação de um instrumento capaz de avaliar com maior abrangência as habilidades esperadas. Somado a isso, realizou-se revisão integrativa de literatura, objetivando compreender as evidências do fenômeno avaliativo na formação do enfermeiro, em que as buscas foram realizadas nas seguintes bases de dados: Literatura Latino-Americana e do Caribe em Ciências da Saúde, Scopus, PubMed, *Excerpta Medica dataBASE* e *Education Resources Information Center*. A busca ocorreu no período entre abril e maio de 2023. Inicialmente, foram encontrados 3.598 artigos, posteriormente, os mesmos foram analisados conforme os critérios de inclusão e exclusão, e 82 artigos compuseram a amostra final da revisão. Subsequentemente, foram consultados documentos oficiais do MEC, Conselho Federal de Enfermagem (COFEN) e Associação Brasileira de Enfermagem^(21-23)^. Destaca-se que, além das DCN-Enf vigentes, também foram utilizadas, para a elaboração do instrumento, as minutas das novas DCN-Enf. De posse destes dados, realizou-se a elaboração da primeira versão dos domínios e itens do IAFEE-ECS^®^.

Posterior a elaboração do IAFEE-ECS^®^, iniciou-se a etapa de validação com o painel de especialistas. A pesquisa foi realizada de forma *online*, buscando participantes de diferentes regiões do país, a fim de possibilitar uma ampla abrangência de especialistas - pesquisadores enfermeiros e coordenadores de curso e/ou docentes diretamente envolvidos no ECS -, pois caracteriza-se como o primeiro passo para a disseminação do conhecimento em desenvolvimento. Ampliar o cenário de coleta de dados em estudos de validação apresenta relevância à medida que diferentes pessoas, com suas percepções, contextos de vida e trabalho, são incluídas em uma amostragem.

Esta etapa foi realizada no período de junho a setembro de 2023 em duas rodadas. Foram enviados convites para 260 especialistas via e-mail contendo breve resumo da pesquisa, seus objetivos, seu método e *link* para acesso ao Termo de Consentimento Livre e Esclarecido. Aceitaram participar do estudo 67 especialistas, que foram informados sobre o prazo estabelecido para retorno do instrumento de validação, que indicaram novos especialistas que atendessem aos critérios de seleção do estudo. Após o aceite, os participantes receberam e-mail com o *link* para acesso ao formulário do *Google Forms* ^®^ contendo o IAFEE-ECS^®^, bem como orientações para realizar sua validação.

Na primeira rodada, 24 especialistas retornaram a validação do instrumento, apresentando sugestões de melhorias. Para cada bloco do IAFEE-ECS^®^, foram avaliados os critérios de clareza, relevância, objetividade, precisão, variedade, modalidade, comportamento, amplitude, equilíbrio, tipicidade, credibilidade e simplicidade^(24)^. O instrumento foi apresentado sob forma de escala do tipo Likert, composta por quatro pontos: 1 - Inadequado; 2 - Pouco Adequado; 3 - Adequado; 4 - Totalmente Adequado. Para os itens avaliados com escore 1 ou 2, solicitou-se que os especialistas fornecessem sugestões de alterações e/ou adição ou exclusão de itens.

Após reuniões de consenso entre as autoras, os blocos avaliativos do IAFEE-ECS^®^ que apresentaram Índice de Validade de Conteúdo (IVC) inferior a 0,80 foram excluídos, assim como outros itens foram alterados conforme opinião dos especialistas. Posteriormente, deu-se início à segunda rodada, por meio de *feedback* controlado aos especialistas que participaram na primeira rodada.

Na segunda rodada, o IAFEE-ECS^®^ revisado foi disponibilizado por meio de *link* do *Google Forms* ^®^, indicando os itens excluídos ou reformulados. Ao conteúdo excluído, foi solicitado que os especialistas indicassem a concordância ou não dessa exclusão. Já os blocos reformulados foram avaliados segundo os mesmos critérios psicométricos da rodada anterior. Destaca-se que não foram incluídos novos participantes nesta rodada. Os especialistas obtiveram um prazo de 20 dias para responder à segunda rodada. Aos que não retornaram, foram encaminhados lembretes semanais durante 15 dias, sendo finalizada a coleta de dados da segunda rodada contando com a participação de 12 especialistas.

### Análise dos resultados e estatística

Os dados foram armazenados em planilhas, sendo analisados com o apoio do programa estatístico *Statistical Package for the Social Sciences*. A avaliação da confiabilidade e consistência interna do instrumento foi analisada a partir do coeficiente do alfa de Cronbach^(25)^. Foi estabelecida a concordância superior a 80% para cada bloco e item submetido à validação^(26)^. Destaca-se que não existe um acordo universal sobre qual deve ser o nível de consenso para um estudo Delphi, devendo ser uma decisão do pesquisador. Assim, a validação global dos critérios psicométricos, bem como dos blocos e itens do instrumento, foi avaliada pelo IVC. No presente estudo, foi considerado adequado IVC ≥ 0,80^(16)^. No processo de análise, também foram considerados os comentários e sugestões dos especialistas, na perspectiva de inclusão de elementos qualitativos, especialmente com o intuito de fornecer informações compreensíveis do instrumento.

## RESULTADOS

O instrumento foi elaborado baseado nos elementos teóricos de avaliação formativa propostos pelo referencial de Perrenoud^(12)^, que tem como objetivo possibilitar a condução da avaliação durante o ECS, podendo ser utilizado pelo professor, enfermeiro supervisor e estudante, viabilizando sua autoavaliação ao longo do período. Para tanto, o instrumento deverá servir como um meio para individualizar o percurso do processo de ensino-aprendizagem ao longo do estágio.

A primeira versão do IAFEE-ECS^®^ apresentou uma estrutura composta por cinco blocos de avaliação: 1 - cabeçalho com dados de identificação do estudante, IES e local de estágio; 2 - apresentação e orientações para a utilização do instrumento; 3 - os quatro domínios do instrumento com seus respectivos itens: I - domínio: competências e habilidades transversais identificadas/evidenciadas no desenvolvimento do estágio curricular supervisionado, contendo nove itens; II - domínio: assistência e cuidado de enfermagem na atenção à saúde humana, contendo dez itens; III - domínio: gestão de enfermagem nos serviços, contendo seis itens; IV - domínio: educação, investigação/pesquisa em enfermagem e saúde, contendo cinco itens; 4 - abaixo de cada domínio, foi disponibilizado espaço para realização de parecer descritivo tanto pelo avaliador quanto pelo estudante, objetivando sua autoavaliação; 5 - ao final do IAFEE-ECS^®^, foi disponibilizada uma fórmula para realizar a avaliação formativa final.

Nas colunas do instrumento, localizadas à direita, o avaliador tem a oportunidade de, a cada avaliação formativa, atribuir nota (pontuação) de 0 a 10 para cada item, considerando o desenvolvimento do estudante no campo de estágio, sendo o mesmo processo realizado pelo estudante em sua autoavaliação. Ao final de cada domínio, deve ser registrada a soma das pontuações de cada item. A soma das notas obtidas em cada domínio, dividida pelo número de itens do instrumento, será o resultado de cada avaliação formativa (AF1 e AF2). Para a obtenção da avaliação somativa do estudante, deverá ser realizada a média aritmética simples das avaliações formativas. Para tanto, deve ser realizada a soma das notas obtidas nas avaliações formativas 1 e 2 (AF1 + AF2) e o resultado dividido por 2, resultando na média final.

Participaram da validação de conteúdo do IAFEE-ECS^®^ 24 especialistas com idade entre 33 e 68 anos, sendo 22 (91,7 %) do sexo feminino e dois (8,3 %) masculino. Todos os participantes possuíam graduação em enfermagem; destes, 15 (62,5%) tinham doutorado, sete (29,2%), pós-doutorado, e dois (8,3%), mestrado. Quanto à experiência profissional, todos estão atuando na docência de ensino superior de enfermagem, porém 23 (95,8%) relataram experiência prévia assistencial, e 14 (58,3%), em cargos de gestão. Em relação às áreas de atuação na pesquisa, 13 (54,2%) tinham especialização em enfermagem médico-cirúrgica, seis (25%), em enfermagem de saúde pública, quatro (16,7%), em enfermagem obstétrica, e um (4,2%), em enfermagem de doenças contagiosas.

Com relação à caracterização do painel de especialistas, houve predominância de especialistas com doutorado, com experiência profissional prévia na assistência e que já atuaram e/ou atuam junto à disciplina do ECS.

Na primeira rodada, participaram 24 especialistas. O *Scale-level Content Validity Index* (S-CVI) dos critérios psicométricos foi de 0,89, e o alfa de Cronbach, de 0,993 (Tabela 1). Não houve consenso entre os especialistas com relação ao título do domínio I (IVC 0,79) e também com relação aos itens 13 (IVC 0,73), 18 (IVC 0,79) e 30 (IVC 0,79) (Tabela 1). Para dar sequência à segunda rodada da validação, a redação do título do domínio I foi alterada; o item 13 foi excluído por estar contemplado na redação do item anterior; e os itens 18 e 30 foram excluídos conforme sugerido pelos especialistas, devido à impossibilidade de utilizá-los na avaliação da prática dos estudantes.

**Tabela 1:** Itens que compõem o Instrumento de Avaliação Formativa de Discentes de Enfermagem em Estágio Curricular Supervisionado, Santa Maria, Rio Grande do Sul, Brasil, 2023

ITENS AVALIADOS			
	IVC		
Elementos de identificação	Rodada 1	Rodada 2	ALFA
Título do instrumento.	0,91	-	0,932
Dados de identificação.	0,89	1,00	0,912
**Itens do instrumento**			
**Domínio I: Competências e habilidades transversais identificadas/evidenciadas no desenvolvimento do estágio curricular supervisionado**	0,79	1,00	0,977
Demonstra domínio do conhecimento teórico e científico sobre os temas que emergem das situações práticas vivenciadas no campo de estágio.	0,85	-	0,964
Demonstra conhecimento das principais políticas e legislações de saúde.	0,83	-	0,966
Compreende a ética e os princípios bioéticos, respeitando a dignidade, a autonomia e a privacidade dos indivíduos.	0,97	-	0,918
Compreende as diversidades de gênero, raciais, culturais, sociais e econômicas dos indivíduos e comunidades.	0,96	-	0,934
Capacidade para trabalhar em ambientes complexos e dinâmicos, adaptando-se a mudanças e novas situações, sendo capaz de resolver problemas que surgem no cotidiano do trabalho.	0,91	-	0,955
Demonstra autonomia na prática de enfermagem dentro dos limites de sua formação e competência.	0,97	-	0,932
Trabalha de forma colaborativa com a equipe de enfermagem e interprofissional.	0,97	-	0,952
Comunica-se, de forma clara, efetiva e respeitosa, com pacientes, familiares e profissionais de saúde, sendo capaz de utilizar formas alternativas de comunicação.	0,96	-	0,938
Demonstra habilidades na execução e registro do Processo de Enfermagem, apoiado nos Sistemas de Linguagem Padronizada de enfermagem.	0,93	-	0,963
**Domínio II: Assistência e cuidado de enfermagem na atenção à saúde humana**	0,88	-	0,978
Demonstra conhecimento nos principais procedimentos, técnicas e tecnologias utilizados na prática de enfermagem.	0,97	-	0,934
Demonstra habilidades na execução de procedimentos e técnicas seguras, além do uso de tecnologias no cuidado.	0,88	-	0,974
Identifica as patologias e condições de saúde prevalentes na população, (co)relacionando aos determinantes sociais de saúde.	0,84	-	0,954
Demonstra reconhecer o processo de saúde-doença prevalentes	0,73	-	0,977
Desenvolve a prática de enfermagem pautada no pensamento crítico, raciocínio clínico e baseado em evidências.	0,94	-	0,959
Presta assistência de enfermagem de forma segura e eficaz.	0,89	-	0,976
Demonstra responsabilidade e compromisso com a melhoria contínua da qualidade do cuidado.	0,95	-	0,919
Presta cuidados de enfermagem compatíveis com as diferentes necessidades apresentadas pelo indivíduo, família e comunidade.	0,96	-	0,984
Compreende a natureza humana em suas dimensões e fases evolutivas.	0,77	-	0,985
Demonstra reconhecer o funcionamento da Rede de Atenção à Saúde, em seus diferentes níveis, na perspectiva da integralidade da assistência.	0,90	-	0,986
**Domínio III: Gestão de enfermagem nos serviços**	0,99	-	0,948
Realiza análise situacional do processo de trabalho para planejar e propor intervenções.	0,93	-	0,990
Organiza e direciona recursos materiais, dimensionamento de pessoal e de infraestrutura para a realização do trabalho de enfermagem.	0,97	-	0,928
Atua junto à equipe de enfermagem e multiprofissional com relacionamento interpessoal adequado, comunicando-se de modo efetivo, interagindo e propondo resolução diante dos problemas encontrados no campo profissional.	0,92	-	0,970
Utiliza instrumentos que qualificam o cuidado (escala de dimensionamento de pessoal, escalas para avaliação da complexidade assistencial, escalas de avaliação da condição atual e/ou de risco do paciente).	0,89	-	0,984
Utiliza o Processo de Enfermagem, incluindo os Sistemas de Linguagem Padronizadas de enfermagem.	0,91	-	0,996
Desenvolve ações de liderança da equipe de enfermagem, promovendo integração, atualização e qualificação.	0,88	-	0,968
**Domínio IV: Educação, investigação/pesquisa em enfermagem e saúde**	0,98	-	0,928
Disposição para o aprendizado contínuo e busca por atualização. Durante o estágio o aluno realizou cursos e/ou participou de eventos.	0,85	-	0,982
Utiliza novas tecnologias de educação, informação e comunicação com a equipe de saúde e/ou pacientes e família.	0,85	-	0,983
Capacidade de planejar, implementar e avaliar estratégias educativas em saúde pautadas no diálogo e valorização dos saberes dos envolvidos.	0,87	-	0,979
Capacidade de elaborar, executar e avaliar projetos de intervenção profissional.	0,80	-	0,987
Planeja, implementa e avalia os programas de formação e qualificação dos trabalhadores.	0,75	-	0,991
**Elementos orientadores para utilização do instrumento**			
Considerando: a apresentação e orientações sobre o uso do instrumento	0,86	1,00	0,955
Orientações e organização da valoração avaliativa para obtenção da média final	0,83	1,00	0,967
Considerando o campo “autoavaliação” (destinado ao discente), localizado ao final de cada domínio.	0,84	1,00	0,961
Considerando o campo “observações/comentários” (destinado ao docente e preceptor), localizado ao final de cada domínio.	0,96	1,00	0,917
Quanto ao fechamento do instrumento.	0,84	1,00	0,985
**S-CVI/Ave**	**0,89**	**0,92**	
**Alfa de Cronbach do instrumento**			**0,993**

*IVC - Índice de Validade de Conteúdo; S-CVI/AVE - Scale-level Content Validity Index/Average.*

Na segunda rodada de avaliação, o S-CVI do instrumento obteve 0,92. Observa-se que não foram apresentados IVC e alfa de Cronbach por critério psicométrico na segunda rodada Delphi, pois foram submetidos à avaliação dos especialistas apenas os itens com IVC inferior a 0,80 (Tabela 2).

**Tabela 2:** Índices de Validade de Conteúdo e confiabilidade do Instrumento de Avaliação Formativa de Discentes de Enfermagem em Estágio Curricular Supervisionado com base nos critérios psicométricos, Santa Maria, Rio Grande do Sul, Brasil, 2023

Critérios psicométricos	IVC	Alfa de Cronbach
	Delphi 1	Delphi 1
Clareza	0,83	0,823
Relevância	0,93	0,895
Objetividade	0,83	0,963
Precisão	0,84	0,962
Variedade	0,91	0,921
Modalidade	0,92	0,935
Comportamento	0,89	0,967
Amplitude	0,91	0,921
Equilíbrio	0,91	0,926
Tipicidade	0,90	0,955
Credibilidade	0,93	0,938
Simplicidade	0,88	0,955
S-CVI	**0,89**	**0,993**

*IVC - Índice de Validade de Conteúdo; S-CVI - Scale-level Content Validity Index.*

Para tanto, no que se refere à confiabilidade do IAFEE-ECS^®^, sua consistência interna, por meio do coeficiente alfa de Cronbach, obteve valores superiores a 0,823 nos 12 critérios psicométricos do instrumento, demonstrando consistência interna elevada (Tabela 2).

De acordo com os comentários e sugestões dos especialistas, houve alterações pontuais nos seguintes blocos: dados de identificação (foram adicionados o campo de “matrícula”, campo para marcar uma das três seguintes opções - “Atenção Primária à Saúde”, “Atenção Secundária à Saúde” e “Atenção Terciária à Saúde” - e um campo destinado ao preenchimento da “carga horária realizada”); ajustes na redação do texto de “apresentação e orientações sobre o uso do instrumento” e no texto de “aplicação do instrumento”, no qual foi adicionada uma breve apresentação de cada domínio; o campo destinado para “autoavaliação”, bem como o “campo para descrever observações/comentários referentes à 1ª e 2ª avaliação formativa, se houver necessidade”, que encontravam-se ao final de cada domínio com apenas um espaço para preenchimento, que foram realocados para o final do instrumento e subdivididos em dois momentos avaliativos, conforme sugerido pelos especialistas, visando acompanhar a evolução do estudante; no fechamento do instrumento, foi incluído um campo para assinalar quem participou da avaliação formativa, como “professor orientador”, “enfermeiro supervisor” e “estudante”, visto que o mesmo poderá ser utilizado tanto pelo estudante, para autoavaliação, quanto pelo professor orientador e também pelo enfermeiro preceptor.

Após o processo de validação com adequação do instrumento conforme a opinião dos especialistas, foi estabelecida sua versão final. Posteriormente, o IAFEE-ECS^®^ foi submetido ao Registro de Direitos Autorais pela Câmara Brasileira do Livro, recebendo ISBN nº 978-65-00-93619-3. A referida versão encontra-se disponível no *link:* https://www.ufsm.br/cursos/pos-graduacao/santa-maria/ppgenf/produtos-tecnico-tecnologicos.

## DISCUSSÃO

Com vistas a uma pedagogia mais eficaz, é indispensável saber quais as competências e habilidades que se pretende levar aos alunos, bem como os caminhos necessários. Soma-se a isso a necessidade de discriminar quais meios se possui para observar os domínios atingidos ou, em vias de aquisição, os métodos de trabalho, as atitudes, os funcionamentos mentais e como se pretende intervir junto aos estudantes por meio de regulações da aprendizagem. Os meios de ensino não são, em sua maioria, concebidos por uma pedagogia diferenciada resultante de uma avaliação formativa. Para ir nesse sentido, deve-se, então, aceitar um grande investimento na criação ou adaptação de instrumentos didáticos^(12)^.

As DCN-Enf destacam que a avaliação formativa dos estudantes deve se basear nos conteúdos curriculares, nas habilidades e competências, buscando o desenvolvimento da crítica, reflexão, ética, entre outros elementos fundamentais^(1)^. Na perspectiva de Perrenoud, a avaliação não é um objetivo em si, mas um meio de verificar se os estudantes adquiriram os conhecimentos necessários e, a partir disso, gerir as situações didáticas. No entanto, cabe salientar que a aprendizagem nunca é linear, pois é procedida por ensaios, tentativas e erros, sugestões e contra-sugestões, explicações complementares, revisão das noções de base, e trabalho sobre o sentido da tarefa ou autoconfiança^(12)^. Com base nisso, a formação de enfermeiros deve possibilitar o desenvolvimento de competências transversais, de modo a preparar profissionais não apenas para uma prática profissional eficiente, mas também para os desafios dinâmicos e multifacetados dos serviços de saúde.

O conhecimento sobre as políticas e legislações de saúde, ética e bioética, e a compreensão das diversidades de gênero, raciais, culturais, sociais e econômicas dos indivíduos e comunidades são elementos fundamentais e interconectados que se encontram presentes na atuação cotidiana do enfermeiro, estando previstos nas DCN-Enf^(1)^. Ao se deparar com acontecimentos reais, o estagiário é impulsionado a refletir sobre as diferentes situações que se apresentam no contexto do trabalho em saúde, conduzindo-o a planejar suas ações para, apenas posteriormente, implementá-las^(27)^. Visto isso, a formação necessita não apenas do entendimento teórico desses elementos, mas também da aplicação da compreensão na interação dos mesmos diante da prática clínica com pacientes e equipes interdisciplinares.

Entre as habilidades e competências específicas das DCN-Enf, está inclusa a capacidade de diagnosticar e solucionar problemas de saúde, de comunicar-se, de tomar decisões, de intervir no processo de trabalho, de trabalhar em equipe e de enfrentar situações em constante mudança^(1)^. Estabelecer diálogo com pacientes e equipes de saúde ou expressar opiniões de forma coerente são dificuldades enfrentadas pelos estudantes durante sua formação, o que ressalta a necessidade de fortalecer as habilidades de comunicação^(28)^. Para tanto, o profissional, para atuar de modo efetivo, deve possuir habilidades interpessoais sólidas, promovendo relacionamentos saudáveis com a equipe multiprofissional e propor soluções diante dos desafios profissionais.

As competências transversais buscam avaliar se o estudante consegue interrelacionar teoria e prática para a execução do Processo de Enfermagem (PE)^(29)^. Na formação, o desenvolvimento da crítica e reflexividade do estudante é fundamental para que ele identifique patologias, condições de saúde, prevalência, incidência, trace indicadores e relacione todos esses achados da sua prática com os determinantes sociais de saúde nos diferentes níveis de atenção, incluindo primária, secundária e terciária.

Avaliação formativa coloca à disposição do professor informações mais precisas e mais qualitativas sobre os processos de aprendizagem, as atitudes e aquisições dos estudantes^(12)^. Para a execução do PE, docente e supervisor devem auxiliar o estudante a ampliar sua visão sobre as condições de saúde e adoecimento da população. Durante este processo de aprendizagem, avaliam-se o pensamento crítico do estudante, seu raciocínio clínico, bem como o potencial para aliar todos os achados da prática clínica com as melhores evidências científicas.

A avaliação formativa se caracteriza como um dispositivo de individualização do processo de formação e de diferenciação das intervenções com cada estudante, buscando valorizar suas necessidades específicas, constituindo uma intervenção diferenciada. Com isso, a avaliação jamais poderá ser analisada em si mesma, mas como componente de um sistema de ação^(12)^. O IAFEE-ECS^®^ traz essa proposição, na qual a enfermagem necessita não só avaliar o saber técnico, mas principalmente avaliar o potencial de cientificidade do estudante. Experimentar a prática deve ir além de identificar necessidades, pois é o momento de utilizar a prática baseada em evidências para desenvolver um planejamento de enfermagem visando implementar condutas seguras e eficazes de cuidado para indivíduo, família e comunidades. Soma-se a isso o (re)conhecimento da Rede de Atenção à Saúde, bem como uma visão a partir da integralidade do cuidado.

O IAFEE-ECS^®^ foi desenvolvido buscando trazer elementos para avaliar habilidades relacionadas à avaliação, ao planejamento, à implementação de intervenções, organizar e direcionar recursos materiais, dimensionamento de pessoal e infraestrutura voltados à qualificação do cuidado. Entretanto, a formação possui fragilidades no desenvolvimento de competência da gestão, sendo pouco aprofundada nas disciplinas ao longo do curso ou abordadas em situações pontuais e isoladamente nos currículos. Gerenciar o cuidado, a equipe de enfermagem e os serviços de saúde não é tarefa fácil, pois desafia o enfermeiro diariamente. Para tanto, identifica-se que os graduandos reconhecem a importância da gestão na formação e salientam que o tema deveria ser abordado desde o primeiro ano da graduação^(30)^.

A avaliação formativa permite ao docente e supervisor uma ideia mais precisa a respeito do funcionamento intelectual do estudante, bem como suas formas de aprender e seu nível de competência^(12)^. Nesse sentido, é indispensável que o profissional esteja em constante atualização para que sustente cientificamente o seu processo de trabalho. Com isso, a utilização de instrumentos qualificadores do cuidado, como escalas de dimensionamento de pessoal e sistemas de classificação, é indispensável, mas sua efetividade requer uma compreensão aprofundada do contexto em que são aplicados. A aplicação do PE reflete a maturidade profissional, mas a eficácia dessas práticas reside na integração coesa com a realidade clínica.

Os estagiários reconhecem a importância do comprometimento pessoal durante o ECS, visto que o desenvolvimento do seu processo decisório requer a mobilização de atributos necessários ao raciocínio crítico e ao enfrentamento de dilemas para decidir o que se julga melhor para o sujeito e para a sociedade a qual pertence^(27)^. A formação do enfermeiro, pautada no aprendizado contínuo, é um pilar crucial diante da dinâmica evolutiva da área da saúde. Nesse sentido, a busca por qualificação (cursos, participação em eventos, atividades de pesquisa e extensão) indica o interesse pessoal em se desenvolver e profissional para se manter atualizado.

A incorporação de novas tecnologias na comunicação com a equipe e pacientes, embora essencial, demanda uma abordagem estratégica que vai além de uma mera inclusão digital. A capacidade de planejar, implementar e avaliar estratégias educativas em saúde, fundamentadas no diálogo e valorização dos saberes, é uma competência determinante. No entanto, sua efetividade está intrinsecamente ligada à compreensão profunda das necessidades específicas do cenário de trabalho. A habilidade de elaborar, executar e avaliar projetos de intervenção profissional é, indubitavelmente, relevante, mas exige que essas iniciativas não se limitem ao plano conceitual, alcançando impacto efetivo na prática clínica.

Muitas vezes, apenas ao final de um ciclo de estudos, a avaliação ocorre, resultando em curtos períodos de prazos para efetivamente auxiliar o estudante em sua aprendizagem, ocorrendo decisões de curto prazo, por vezes, dificilmente reversíveis^(12)^. No que tange ao uso do IAFEE-ECS^®^ pelos docentes, enfermeiros supervisores e estudantes, recomenda-se que não ocorra somente ao final do processo avaliativo, com o intuito somente de atribuir nota ao estudante. Assim, recomenda-se que seu uso acompanhe o processo avaliativo desde o início do ECS, no sentido de servir como uma ferramenta para a avaliação diagnóstica contínua da aprendizagem. A adoção dessa sistemática pode contribuir para identificação de problemas e dificuldades, e monitoramento do processo de formação por meio de *feedback* do desempenho dos estudantes, permitindo intervenções para a melhoria da aprendizagem.

É importante formar os estudantes para a regulação de seus próprios processos de pensamento e aprendizagem^(12)^. O instrumento também poderá ser utilizado para autorregulação, oportunizando ao estudante olhar para suas competências e habilidades e planejar, em conjunto com seu enfermeiro supervisor e docente, sua evolução contínua.

Adotar a avaliação da aprendizagem sob a ótica formativa instiga o desenvolvimento de uma reflexão sobre alternativas à avaliação tradicional, especialmente no sentido de uma observação baseada em objetivos ou critérios de domínio do que se pretende avaliar. Embora nada se transforme de um dia para o outro no processo formativo, (re)pensar a avaliação na formação do enfermeiro também instiga a IES a reconsiderar todo o processo de ensino, tendo em vista que ambos estão diretamente correlacionados.

Neste estudo, consideraram-se os estudantes como o centro do processo de ensino da prática clínica, dando plena consideração ao estilo de aprendizagem individual. Poderia garantir o interesse de aprendizagem do estudante de enfermagem e melhorar o efeito de recebimento de conhecimento^(7)^. É importante esse *feedback* entre estudantes, supervisores, docentes e instituição de ensino, para que o processo avaliativo atinja seu objetivo como estratégia de melhoria do desempenho dos estudantes^(31)^.

### Limitações do estudo

A pesquisa se limita por incluir participantes de Instituições Públicas de Ensino Superior em enfermagem, não sendo contempladas instituições privadas.

### Contribuições para a área da enfermagem

Como contribuição para a enfermagem, acredita-se que um instrumento baseado nas DCN-Enf, documentos oficiais do COFEN, bem como na literatura nacional e internacional, sustentado em um referencial teórico da área da educação que defende uma pedagogia diferenciada baseada em uma avaliação formativa, pode servir como um meio para subsidiar a avaliação da aprendizagem no decorrer do ECS, podendo ser utilizada tanto pelo docente quanto pelo enfermeiro supervisor e pelo próprio estudante, para sua autorregulação.

## CONCLUSÕES

O IAFEE-ECS^®^, após sua validação com especialistas, apresenta domínios, itens e elementos orientadores para seu uso, demonstrando sua possibilidade para uma avaliação formativa, processual e autorreguladora de estudantes que estão realizando o ECS. O estudo tem potencial para impulsionar novas discussões acerca dos processos avaliativos não só no período do ECS, mas no decorrer da formação do enfermeiro, sob a ótica de uma pedagogia inovadora que inclua a avaliação no centro dos processos pedagógicos com o intuito de servir como um meio para regular as aprendizagens e não somente como uma avaliação pontual com o objetivo de cumprir com a obrigatoriedade de dar notas ao estudante. Recomenda-se a aplicação do instrumento em diferentes cenários, incluindo atenção primária, secundária e terciária, por todos os atores envolvidos no ECS, visando verificar sua aplicabilidade na prática do estágio.

## Data Availability

Os dados de pesquisa só estão disponíveis mediante solicitação.
